# Safety and immunogenicity of the quadrivalent human papillomavirus (qHPV) vaccine in HIV-positive Spanish men who have sex with men (MSM)

**DOI:** 10.1186/s12981-017-0160-0

**Published:** 2017-07-18

**Authors:** Carmen Hidalgo-Tenorio, Jessica Ramírez-Taboada, Concepción Gil-Anguita, Javier Esquivias, Mohamed Omar-Mohamed-Balgahata, Antonio SamPedro, Miguel Lopez-Ruz, Juan Pasquau

**Affiliations:** 1 Infectious Disease Service, University Hospital Virgen de las Nieves, Granada, Spain; 2 Pathology Service, University Hospital Virgen de las Nieves, Granada, Spain; 3 Infectious Disease Unit of the Hospital Ciudad de Jaén, Jaén, Spain; 4 Microbiology Service, University Hospital Virgen de las Nieves, Granada, Spain

**Keywords:** Quadrivalent HPV vaccine, High squamous intra-epithelial lesions (HSIL), Low squamous intra-epithelial lesion (LSIL), Human immunodeficiency virus (HIV), Men having sex with men (MSM), Anal cancer

## Abstract

**Background:**

Safety and immunogenicity of the quadrivalent human papillomavirus (qHPV) vaccine were evaluated in HIV-positive Spanish MSM. The prevalence of High Squamous Intraepithelial Lesions (HSIL) and genotypes of high-risk human papillomavirus (HR-HPV) were also determined, as well as risk factors associated with the presence of HR-HPV in anal mucosa.

**Methods:**

This is a randomised, double blind, placebo-controlled trial of the quadrivalent HPV (qHPV) vaccine. The study enrolled from May 2012 to May 2014. Vaccine and placebo were administered at 0, 2 and 6 months (V1, V2, V3 clinical visits). Vaccine antibody titres were evaluated at 7 months. Cytology (Thin Prep^®^ Pap Test), HPV PCR genotyping (Linear Array HPV Genotyping Test), and high-resolution anoscopy (Zeiss 150 fc© colposcope) were performed at V1.

**Results:**

Patients (n = 162; mean age 37.9 years) were screened for inclusion; 14.2% had HSIL, 73.1% HR-HPV and 4.5% simultaneous infection with HPV16 and 18. Study participants (n = 129) were randomized to qHPV vaccine or placebo. The most common adverse event was injection-site pain predominating in the placebo group [the first dose (83.6% vs. 56.1%; p = 0.0001]; the second dose (87.8% vs. 98.4%; p = 0.0001); the third dose (67.7% vs. 91.9%; p = 0.0001). The vaccine did not influence either the viral load of HIV or the levels of CD4. Of those vaccinated, 76% had antibodies to HPV vs. 30.2% of those receiving placebo (p = 0.0001). In the multivariate analysis, Older age was associated with lower HR-HPV infection (RR 0.97; 95% CI 0.96–0.99), and risk factor were viral load of HIV >200 copies/µL (RR 1.42 95% CI 1.17–1.73) and early commencement of sexual activity (RR 1.35; 95% CI 1.001–1.811).

**Conclusions:**

This trial showed significantly higher anti-HR-HPV antibody titres in vaccinated individuals than in unvaccinated controls. There were no serious adverse events attributable to the vaccine. In our cohort, 1 of every 7 patients had HSIL and the prevalence of combined infection by genotypes 16 and 18 was low. This suggests that patients could benefit from receiving qHPV vaccine. Older age was the main protective factor against HR-HPV infection, and non-suppressed HIV viremia was a risk factor.

Clinical trial registration: ISRCTN14732216 (http://www.isrctn.com/ISRCTN14732216).

## Background

Anal squamous cell carcinoma (ASCC) in HIV patients is, currently, one of the most-frequent non-AIDS-defining cancers [1]. There are several studies that confirm its higher prevalence in HIV-positive individuals compared to the seronegative population; in one of them, the prevalence of anal HPV infection was 60% among HIV-negative men who have sex with men (MSM) and 93% among HIV-positive MSM [2]. However, its special relevance in HIV patients is not only due to its high prevalence, in MSM and women with cervical pathologies [3, 4], but also because of its greater rapidity of progression if not treated early. In a study carried out in Seattle, Washington, in MSM who were free of Anal intraepithelial neoplasia, High Squamous Intraepithelial Lesion (HSIL) developed in 15% of HIV-positive and 8% of HIV-negative men in an average of 21 months [5]. One of the risk factors implicated in the appearance of pre-malignant lesions (HSIL) and ASCC is the chronic infection by high-risk human papillomavirus (HR-HPV) genotypes [6, 7].

The classical risk factors involved in the infection of oncogenic HPV in the anal mucosa include: young age [8], a large number of sexual partners [9], and in HIV-positive MSM patients, low CD4 counts, among others [10].

Several strategies for the prevention of ASCC have been evaluated, including: screening for ASCC/HSIL with anal cytology alone [11, 12], anal cytology and HPV-PCR [13–18], or high-resolution anoscopy (HRA) [19]; and prevention of HPV infection with condom use [20] and vaccination [21].

There have been several trials with the quadrivalent human papillomavirus (qHPV) vaccine carried-out in HIV seronegative MSM. In two of them, MSM were compared to heterosexual males (HM), and they found lower protection rates against external genital lesions (EGL) in MSM (rate of EGL in MSM 0.42/100 person-year at risk, vs. in HM 0.08/100 person-year at risk) [22], and a lower antibody response against the qHPV vaccine (at month 36: Ab of HPV-6 was in HM 89.5% vs. 80% in MSM; Ab HPV-11 94.3% vs. 89.1%; HPV-16 98.3% vs. 93.9%; HPV-18 57.3% vs. 53.6%) [23]. Another trial conducted only in MSM showed effectiveness in preventing the appearance of HSIL in approximately half of patients [24]. Until now, there have only been 2 clinical trials with the qHPV vaccine carried out in adult HIV+ population, one in MSM in which immunogenicity was found to be close to 100% [25]; and another in men and women that was interrupted due to lack of efficacy against the appearance of anal high-grade squamous intraepithelial lesions in anal mucosa [26]. With these data, we decided to conduct a randomised, single-centred, double blind trial of the qHPV vaccine in HIV-positive MSM patients. The main objective of this paper was to assess the safety and immunogenic capacity of the vaccine in adult Spanish HIV-positive MSM patients. The secondary objectives were to evaluate the prevalence of high squamous intraepithelial lesion (HSIL) and HR-HPV, as well as the predictive factors associated with the infection by this virus in anal mucosa.

## Patients and methods

### Trial design

 This is a randomised, double blind, placebo-controlled trial of the quadrivalent HPV (qHPV) vaccine. It was conducted according to the protocols of the Spanish Drugs and Health Products Agency (*Agencia Española del Medicamento y Productos Sanitarios; AEMPS*). The recruitment period was between May 15th 2012 and May 15th 2014. Clinical trial registration: ISRCTNregistry. ISRCTN14732216. DOI 10.1186/ISRCTN14732216. Date assigned 02/08/2016. Retrospectively registered. See clinical trial protocol at http://www.isrctn.com/ISRCTN14732216.

### Participants

#### Inclusion criteria

- HIV-positive MSM patients of ≥18 years of age who, at the time of study inclusion were not infected simultaneously by the four genotypes of HPV that the quadrivalent vaccine addresses.
- Patients who had a normal high-resolution anoscopy (HRA) at screening for inclusion or only had condylomas and/or low squamous intraepithelial lesion (LSIL) in anal biopsy.

#### Exclusion criteria

- HIV MSM patients who had simultaneous anal infection with the four genotypes addressed by the vaccine, and who at least had HPV genotypes 16 and 18.
- Active opportunist infection at the time of recruitment into the study.
- Patients who, in screening anoscopy had HSIL, or ASCC or had received treatment for these lesions (these patients were excluded because patients with HSIL have a higher probability of progression to ASCC).
- History of allergy to aluminium and/or yeast extract excipient.

#### Settings and locations

The patients who enrolled were HIV-positive MSM that were attending the Infectious Diseases Service of the “*University Hospital Virgen de las Nieves”, Granada* (Spain), and *Ciudad de Jaén* (Spain).

The purposes of the study were explained to the potential participants who then underwent screening, and enrolled if they met the inclusion criteria for the trial. They were asked to sign the fully informed consent form. The project received approval from the hospital’s Ethics Committee (Institution Review Board) “Comité de Ética de la Investigación Biomédica de la Provincia de Granada (CEI-GRANADA)” University Hospital Virgen de las Nieves. The study was conducted in compliance with ethical and moral principles stated in the Declaration of Helsinki as well as the current Spanish Laws on Biomedical Research. Data were coded to ensure anonymity. The project received funding from the Foundation for Progress and Health (*Fundación Progreso y Salud*) of the Government of Andalucia (2011 convocation). This trial was registered in Clinical trial registration: ISRCTN14732216.

#### Data collection

All data were collected and coded to ensure anonymity according to the current legal requirements in Spain.

At the initial clinical visit (V1), the conditions and objectives of the study were explained. The details were summarised in a document, which was presented to the patient who then signed the informed consent form.

*Clinical-epidemiological variables* At the baseline visit (V1), data collected included: age, number of different partners participating in anal intercourse in the previous 12 months, and over the whole sexual life of the participant; use of prophylactics and percentage frequency of their use; work status (actively employed, unemployed, retired), education level, smoking habit (packets/year), alcohol abuse (standard units of alcohol (SUA) consumed per day); intra-venous drug abuse (IVDA); HIV infection route, months since HIV diagnosis, CDC status, CD4 nadir (considered to be the lowest level of CD4 throughout evolution of HIV), months of anti-retroviral therapy (ART), current line of ART, virological failure (considered to be two consecutives viral load of HIV over 50 copies/µL), concomitant treatment. Other diseases included chronic viral hepatitis B infection (VHB), or hepatitis C (VHC), syphilis, other sexually transmitted diseases (STD), history of anal and/or genital condylomas and the therapy employed, current condylomas, latent active or treated tuberculosis infection.

At clinical visits at 2 and 6 months (V2 and V3), the data collected (again) were number of different anal-sex partners, use of condoms, ART therapy (adherence, change, relapse), appearance of STD or condylomas. The adverse events assessment system employed was a questionnaire that included the most frequent local reactions such as fever, nausea, vomiting, dizziness, syncope, headache and others such as allergic reaction, pruritus, difficulty breathing and/or wheezing. Rare occurrences included lymphadenopathies, chest and lower-limb pain, confusion, chills, muscle pain. The adverse events (AE) were graded on a scale of 1–4. In case of AE grade 4, the blind of the vial administered was broken and, if the code identified the vaccine, the reaction was communicated immediately to the relevant drug-vigilance authorities.

*Blood analyses* At visits V1, V2 and V3 full blood haemogram and blood chemistry analysis were measured, together with CD4, CD8 lymphocytes counts, and HIV viral load (VL).

Antibodies against the 4 genotypes of the qHPV vaccine were determined after the 3rd dose, at 7th month. The analyses were performed in the microbiology department of the hospital by the same microbiologist of the research team (AS). The assays were performed using HPVG ELISA commercial kit (DIA.PRO, Milano, Italy), according to the manufacturer’s instructions. The ELISA kit measures antibodies against the major capsidic protein (L1) of HPV types 6, 11, 16, 18. The blood samples collected in the clinic were centrifuged in the laboratory, frozen at −20 °C and thawed for use at the time of the assay. The results were expressed qualitatively as positive or negative.

*High*-*resolution anoscopy (HRA)* (at V1) All the recruited patients had an anoscopy performed and a biopsy taken following 3% acetic acid instillation and Lugol. Biopsies were taken of acetic-white change and Lugol-negative zones, and normal mucosa, with a minimum of 1 biopsy in each of the four quadrants (right, left, anterior, posterior). The colposcopy equipment employed was Zeiss 150 fc^©^.

*PCR of the HPV and anal cytology* (at V1): 2 mucosa samples were taken from the anal canal with cotton swabs soaked in physiologic saline serum. The samples were stored in liquid medium (Thin Prep^®^ Pap Test) for the detection and genotyping of the HPV using the polymerase chain reaction (PCR) qualitative technique (Linear Array HPV Genotyping Test) performed in a Gene Amp PCR System 9700 thermocycler (Applied Biosystems, Roche, Switzerland). Cytology evaluation was with the Thin Prep^®^ Pap Test (Thin Prep Processor 2000, Hologic Corp, USA). Both samples were sent to the anatomy–pathology laboratory where the same senior pathologist of the research team (JE) carried out the cytology evaluation, validation of PCR methodology, and histology analyses. HPV genotypes 16, 18, 26, 31, 33, 35, 39, 45, 51–53, 56, 58, 59, 66, 68, 73 and 82 were considered high-risk-HPV (HR-HPV). Genotypes 6, 11, 34, 40, 42–44, 54, 55, 57, 61, 70–72, 81, 83, 84 and 89 were considered low-risk-HPV (LR-HPV). The HPV-18 virus species was classified as genotypes 39, 45, 59, 68; and of the HPV-16 species as genotypes 31, 33, 35, 52, 58, 67 [27]. The cytology classification was that of Bethesda [28] which classifies the lesions into 3 types: atypical squamous cells (ASC) (atypical squamous cells undetermined significance [ASC-US], and atypical squamous cells that cannot exclude high grade squamous intraepithelial lesion [ASC-H]), LSIL and HSIL. The histology classification employed was the Squamous Terminology (LAST) Standardization Project for HPV that segregates the lesions as LSIL (AIN1/condyloma), HSIL (AIN2, AIN3), and invasive carcinoma (ASCC) [29].

*Quadrivalent (HPVs 6/11/16/18) vaccine and placebo*: subjects were randomly assigned in a 1:1 ratio to receive vaccine or placebo at day 1, month 2 and month 6. The HPV vaccine was the quadrivalent HPV vaccine (Gardasil©; Merck Research Laboratories). Vaccine or placebo was administered as a 0.5 ml injection in the deltoid muscle (with all three doses administered in the same arm). Placebo was the same quantity (0.5 mL) of excipient provided to us by the hospital’s pharmacy department. The placebo had 0.5 mL of “Solvent for injectable solutions”, this is water used in the preparation of injectable with <1 mmol of Na. All vials were numbered according to patient assignment, and kept until the end of the study.

### Sample size

Based on our own data, 67.2% of HIV MSM patients have a pathological anal biopsy and, of these, 29.8% have HSIL (≥AIN2) with a rate of colonization by high-risk genotypes of 74.2% [30]. To demonstrate a reduction of at least 50% with the administration of the qHPV vaccine, with a statistical power of 80% and a level of significance of 5%, it would be necessary to include 29 patients per group. Nevertheless, we increased the sample to 60 in each arm with an intention to strengthen the statistical power to 98%.

### Randomisation

For allocation of the participants, a computer generated a list of random numbers that was assigned to each patient. Randomization sequence was created using Epidat (Epidat 4.2, 2016. Consellería de Sanidade, Xunta de Galicia, España; Organización Panamericana de la salud (OPS-OMS); Universidad CES, Colombia) statistical software. Participants were randomly assigned following simple randomization procedures to experimental or placebo groups. The person in charge of generating and keeping the list was not part of the research team and did not participate in evaluation or enrollment of patients, therefore guaranteeing patient blinding. This was a double-blind study and randomization was prior to any study interventions.

### Statistical methodology

#### Descriptive analyses

Descriptions of the principal variables collected in the study were, for the quantitative variables, measures of central tendencies and dispersion: mean, standard deviation, median, percentiles, and for the qualitative variables, absolute and relative frequencies. The prevalence with 95% confidence interval (CI) was calculated for HPV and the dysplastic lesions of anal mucosa.

Bivariate analysis was employed to assess the different factors associated with infection by HR-HPV genotypes. For quantitative variables, the Student *t* test for independent variables with a normal distribution, or the Mann–Whitney test in case of non-normal distribution were used to compare means. The χ^2^ test of Pearson or Fisher test was used to compare qualitative variables, depending on the criteria for use. The Kolmogorov–Smirnov test was applied to compare different variables fulfilling the hypothesis of normality of distribution. The FDR correction for multiple comparisons was applied [31].

Multivariate analyses were applied using Poisson regression analysis. Variables that were statistically significant in the bivariate analysis and those that were considered clinically relevant were introduced into the model. The variables were entered into the model using a forward stepwise selection with a probability of between 0.05 and 0.10 for each entry.

All the tests had a probability level of 0.05 for statistical significance. The SPSS package (version 19) and Stata Statistical Software: Release 12 was used throughout.

## Results

### Study population: screening

Between May 15th, 2012 and May 15th, 2014, 162 subjects were screened. Mean age was 37.9 years; CD4 nadir was 337.8 cells/µL; 91.4% of the patients were receiving ART and had a mean CD4 of 688.5 cells/µL; only 3.3% of patients were in virological failure. The epidemiological, clinical and analytical variables are summarised in Table 1.

**Table 1:** Baseline demographic of HIV-positive MSM screened

Variables	HIV-MSM patients n = 162
Mean age; years ±SD	37.9 ± 10.2
Spanish, n (%)	153 (94.2)
Primary school, n (%)	21 (13)
Secondary school; technical school, n (%)	53 (32.7)
University, n (%)	88 (54.3)
Median months of sexual activity (P_25_–P_75_)	216 (120–300)
Median of anal-receptive sexual partners in previous 12 months (P_25_–P_75_)	1 (1–5)
Overall median number of sexual partners, (P_25_–P_75_)	70 (20–250)
Condom users, n (%)	127 (78.4)
Median percentage of condom use, (P_25_–P_75_)	100% (50–100%)
History of condylomatosis, n (%)	47 (29)
Current condylomatosis, n (%)	48 (29.6)
Syphilis treated, n (%)	35 (21.6)
Latent treated tuberculosis, n (%)	10 (11.8)
Chronic HCV infection, n (%)	4 (4.7)
Chronic HBV infection, n (%)	1 (1.2)
Smoking habit, n (%)	33 (38.8)
Ex-smoker, n (%)	12 (14.1)
Ex-IVDA, n (%)	1 (1.2)
Alcohol, median SUA, (P_25_–P_75_)	1 (0–1)
Prior AIDS diagnosis, n (%), 95% CI	27.8 (45)
Median time of HIV duration, months (P_25_–P_75_)	57 (24.5–120)
CD4 nadir, cells/µL, (±SD)	337.8 (±205)
Treatment naïve, n (%)	14 (8.6)
Median ART, months (P_25_–P_75_)	37 (13–88)
Median lines of ART, n (P_25_–P_75_)	1 (1–2)
Virological failure, n (%), 95% CI	5 (3.3)
Baseline CD4, cells/µL (±SD)	688 (±256.4)
Baseline CD8, cells/µL (±SD)	976.5 (±399.4)
Baseline viral load, log10 (±SD)	3.74 (±4.5)

*HCV* hepatitis C virus, *HBV* hepatitis B virus, *LSIL* low-squamous intra-epithelial lesion, *HSIL* high-squamous intra-epithelial lesion, *ASC* indeterminate lesion, *HPV* human papilloma virus, *SUA* standard unit of alcohol, *Ex-IVDA* ex-intravenous drug abuser, *HR-HPV* high risk HPV, *LR-HPV* low risk HPV, *SD* standard deviation, *IQR* interquartile range

In anal mucosa, 26.3% of patients had genotype HPV16, 12.8% HPV18, 17.3% HPV6, 13.5% HPV11, 4.5% had simultaneous infection with oncogenic genotypes 16 and 18. 36.9% had HPV18 species and 50% HPV16 species. Finally, anal mucosa biopsy analysis showed that 40.1% were normal, 45.7% LSIL and 14.2% HSIL (Table 2). The cytology, PCR of HPV and anal biopsy results are summarised in Table 2.

**Table 2:** HPV PCR, cytology and anoscopy variables of the screening population

Variables	n = 162
HPV PCR positive, n (%)	n = 156
LR-HPV	90 (57.7)
HR-HPV	71 (73.7)
HR and LR-HPV	76 (48.7)
Vaccine genotypes, n (%)	
HPV6	27 (17.3)
HPV11	21 (13.5)
HPV16	41 (26.3)
HPV18	20 (12.8)
HPV16 and HPV18	7 (4.5)
HPV of the HPV16 species (31, 33, 35, 52, 58, 67)	80 (50%)
HPV of the HPV18 species (39, 45, 59, 68)	59 (36.9%)
Anal cytology, n (%)	n = 160
Normal	62 (38.8)
LSIL	76 (47.5)
HSIL	9 (5.6)
ASCUS	13 (8.1)
Anoscopy histology, n (%)	n = 162
Normal	65 (40.1)
LSIL	74 (45.7)
HSIL	23 (14.2)
ASCC	0

*LSIL* low squamous intra-epithelial lesion, *HSIL* high squamous intra-epithelial lesion, *ASC-US* atypical squamous cells undetermined significance, *HPV* human papillomavirus, *HR-HPV* high-risk HPV, *LR-HPV* low-risk HPV

**Fig. 1:**
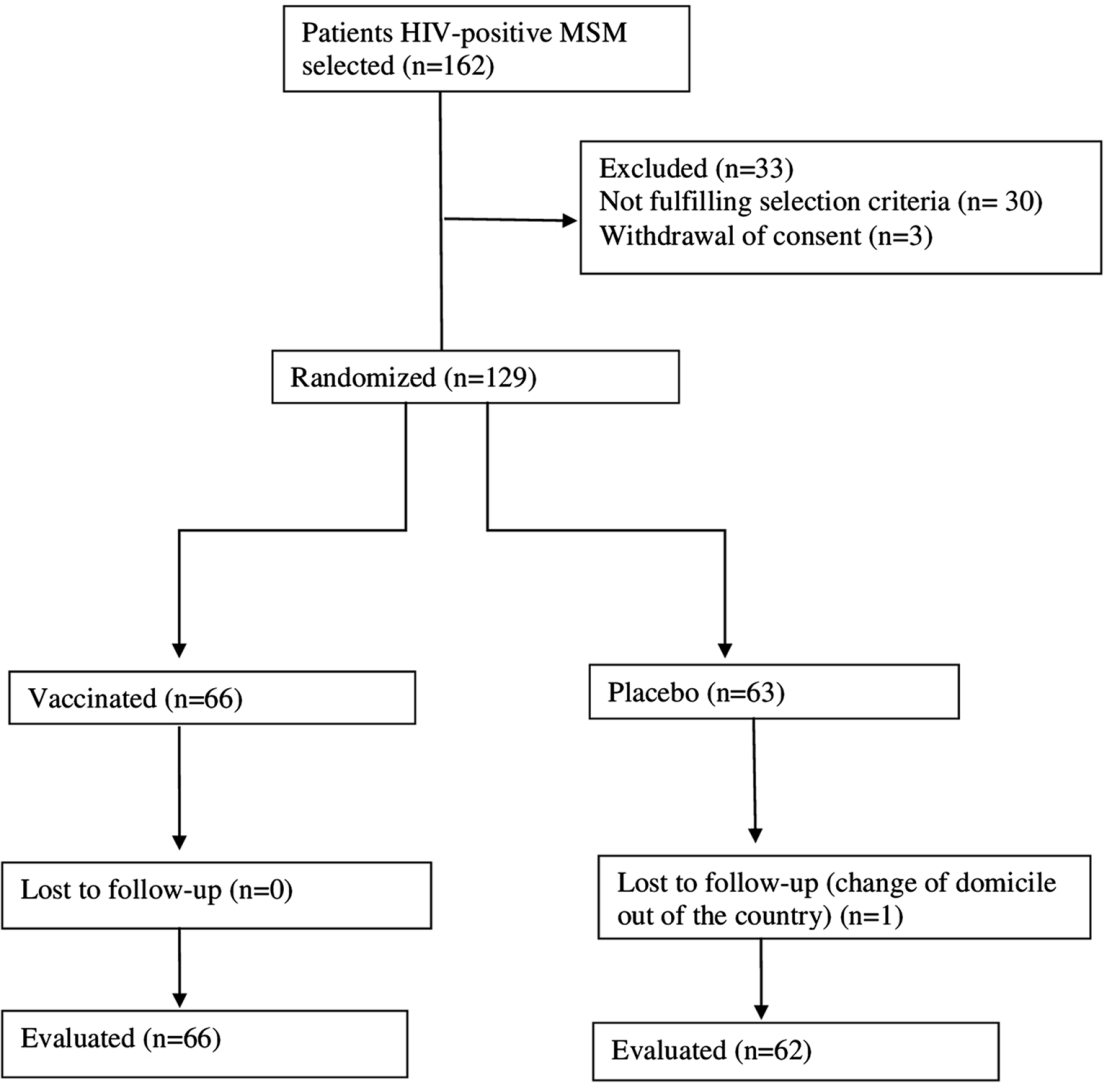
Flow of subjects through the study

Of the 162 patients, 129 (79.6%) were finally included in the trial; 30 (18.5%) were excluded for not fulfilling the selection criteria and 3 (1.8%) for withdrawal of consent; 66 (51.2%) were vaccinated while 63 (48.8%) received placebo; 128 (99.2%) received 3 doses of the vaccine (or placebo) and completed the first 6 months of follow-up. One participant left the country following the 1st vaccination, and was lost to follow-up (Fig. 1).

### Patients enrolled in clinical trial

The patients who received placebo were similar to those who received the vaccine with respect to age, variables related to HIV, ART, others STD, number of lifetime sexual partners, years of sexual relationships and tobacco consumption. The variables are summarised in Table 3.

**Table 3:** Baseline demographics of HIV-positive MSM enrolled in clinical trial

	HIV-MSM vaccine (n = 66)	HIV-MSM placebo (n = 63)	p*
Age, years; mean (±SD)	37.3 (±10.6)	40.5 (±10.02)	0.082
Spanish nationality, n (%)	63 (95.5)	60 (95.2)	0.2
University education, n (%)	34 (51.5)	35 (55.5)	0.56
Partners in the previous 12 months; median (IQR)	1 (1–3)	1 (1–5)	0.8
Life-time partners; n, median (IQR)	50 (20–300)	100 (45–350)	0.041*
Years of sexual activity; median (IQR)	17 (9–24)	21 (13–27)	0.025*
Condom use, n (%)	53 (80.3)	47 (74.6)	0.4
Perianal/genital condylomas at screening, n (%)	20 (30.3)	20 (31.7)	0.86
History of condylomas, n (%)	19 (28.8)	15 (23.8)	0.52
Duration of HIV; mean months (IQR)	58 (26–120)	77 (37–138)	0.2
History of AIDS; n (%)	19 (28.8)	21 (33.3)	0.58
CD4 mean nadir; cells/µL, (±SD)	336 (±227.3)	334.2 (±193.7)	0.96
CD4 mean; cells/µL (±SD)	733 (±252.7)	710.4 (±266.6)	0.62
CD8 mean; cells/µL (±SD)	999.9 (±463.6)	992.2 (±374.9)	0.98
VL of HIV log10; copies/mL (±SD)	3.76 (±4.5)	3.67 (±4.46)	0.8
VL <50 copies/mL, n (%)	53 (80.3)	53 (84.1)	0.57
Virological failure, n (%)	1 (1.5)	3 (4.8)	0.29
Median duration of ART; months (IQR)	42 (17–86)	43 (17–129)	0.42
Number of lines of ART, median (IQR)	1 (1–2)	1 (1–2)	0.56
Syphilis treated, n (%)	16 (24.2)	12 (19.1)	0.47
Other STD, n (%)	11 (16.6)	12 (19.1)	0.72
Latent tuberculosis treated, n (%)	5 (7.6)	10 (15.9)	0.14
HCV, n (%)	2 (3)	2 (3.2)	1
HBV, n (%)	2 (3)	0 (0)	0.49
Smoking, packets/year, median (IQR)	0.2 (0–1)	6.5 (0–18)	0.008*
Ex-smoking, n (%)	10 (15)	13 (20.6)	0.0.42
Ex-IVDA, n (%)	1 (1.5)	0 (0)	0.42
Alcohol (standard units of alcohol; SUA)	0 (0–1)	0.4 (0–1.4)	0.15

p*: p < 0.05

* After applying the FDR correction for multiple comparisons, none of the 3 variables we statistically significant

The group of patients receiving the vaccine and the placebo were similar in relation to the PCR of HPV, cytology and anal histology (Table 4).

**Table 4:** Baseline HPV PCR and HRA results of patients enrolled

Variable	HIV-MSM vaccine (n = 66)	HIV-MSM placebo (n = 63)	p*
PCR of HPV			
LR-HPV, n (%)	39 (59)	34 (53.9)	0.63
HR-HPV, n (%)	46 (69.7)	44 (69.8)	0.87
HR and LR HPV, n (%)	28 (42.4)	31 (49.2)	0.39
Number of HR-HPV (IQR)	1 (0–2)	2 (0–3)	0.22
Number of LR-HPV (IQR)	1 (0–2)	1 (0–2)	0.94
Genotypes, n (%)			
HPV6	11 (16.6)	8 (12.6)	0.55
HPV11	8 (12.1)	7 (11.1)	0.88
HPV16	15 (22.7)	15 (23.8)	0.85
HPV16 species	22 (33.3)	24 (38.1)	0.49
HPV18	4 (6.1)	5 (7.9)	0.74
HPV18 species	18 (27.3)	22 (34.9)	0.35
Cytology, n (%)			
Normal	26 (39.4)	27 (42.9)	0.69
LSIL	34 (51.5)	26 (41.3)	0.24
HSIL	1 (1.5)	4 (6.3)	0.21
ASC	5 (7.6)	6 (9.5)	0.69
HRA, n (%)			
Normal	33 (0.5)	29 (46)	0.65
LSIL	33 (0.5)	34 (53.9)	0.65

*HRA* high resolution anoscopy

* p < 0.05

### Safety of qHPV vaccine

Adverse events (AEs) were higher in the placebo group compared to vaccine group at the time of the first dose (87% vs. 54%; p = 0.0001); the most common being injection-site pain (83.6% vs. 56.1%; p = 0.0001). As for the second dose, there were no significant differences in the frequencies of AEs in general between the Vaccine vs. placebo arms (89.4% vs. 98.4%; p = 0.06), but there were differences once more in regards to pain (87.8% vs. 98.4%; p = 0.0001). Finally, at the third dose, the AEs were higher in the placebo arm again (66.7% vs. 91.9%; p = 0.0001), with pain being significantly greater in this arm (67.7% vs. 91.9%; p = 0.0001). No other AEs were observed. Based on our laboratory values, we observed no patients in either arm with grade 3–4 analytical abnormalities (Table 5).

**Table 5:** On-treatment safety and tolerability

Adverse events	V1: vaccine vs. placebo N (%) p*	V2: vaccine vs. placebo N (%) p*	V3: vaccine vs. placebo N (%) p*
Total AE	36 (54.4) vs. 55 (87.3) 0.0001	59 (89.4) vs. 62 (98.4) 0.06	44 (66.7) vs. 57 (91.9) 0.0001
Injection-site pain	37 (56.1) vs. 53 (83.6) 0.0001	58 (87.8) vs. 62 (98.4) 0.0001	45 (67.7) vs. 57 (91.9) 0.0001
Local itching	10 (15.1) vs. 5 (8) 0.13	1 (1.5) vs. 1 (1.6) 0.37	1 (1.5) vs. 0 0.33
Syncope	2 (3) vs. 0 0.5	1 (1.5) vs. 0 0.33	1 (1.5) vs. 0 0.33
AE leading to treatment discontinuation	0 vs. 0	0 vs. 0	0 vs. 0
Deaths	0 vs. 0	0 vs. 0	0 vs. 0
Serious AE	0 vs. 0	0 vs. 0	0 vs. 0
Grade 3 or 4 abnormalities	0 vs. 0	0 vs. 0	0 vs. 0

Treatment-emergent grade 3 or 4 abnormalities defined by laboratory values: ALT >5.0 × upper limit of normal (ULN); AST >5.0 × ULN; total bilirubin >2.5 × ULN

* p < 0.05

Similarly, there were no statistically significant differences with respect to the levels of CD4 (at V1: vaccine 668 cells/µL vs. placebo 772 cells/µL, p = 0.5; at V2: vaccine 733 cells/µL vs. placeb*o* 692 cells/µL, p = 0.89; at V3: vaccine 705 cells/µL vs. placebo 702 cells/µL, p = 0.94) and HIV viral load [(at V1: median vaccine 0 copies/mL (IQR 0–0) vs. median placebo 0 (IQR 0–20.5), p = 0.38; at V2: median vaccine 0 (IQR 0–0) vs. median placebo 0 (IQR 0–20.5), p = 0.9; and at V3: median vaccine 0 (IQR 0–0) vs. median placebo 0 (IQR 0–10.5), p = 0.98)].

### Immunogenicity of qHPV vaccine

Of those vaccinated, 76% had antibodies (Ab) against HPV at 7 month vs. 30.2% of those receiving placebo (p = 0.0001). There was no record of the baseline (V0/screening) HPV antibody status of the patients.

### Risk factors associated with the presence of HR-HPV genotypes

#### Bivariate analysis

The following variables were observed to be protective factors against the infection of genotypes of HR-HPV in the anal mucosa: age, i.e. being older (without HR-HPV: 42.1 years vs. with HR-HPV 36.3, p = 0.02); duration of ART (without HR-HPV: 60.5 months vs. 28 months; p = 0.008); and months since HIV diagnosis (without HR-HPV: 74 vs. 54; p = 0.02). Conversely, we found the following predictive factors: early age of sexual activity commencement (without HR-HPV: 21 years vs. 17 years; p = 0.017); and HIV viral load ≥200 copies/mL (without HR-HPV: 2.3% vs. 17.9%, p = 0.01) (Table 6).

**Table 6:** Bivariate and multivariable analysis of factors associated with HR-HPV infection

Factors	HIV-MSM with HR-HPV (n = 117)	HIV-MSM without HR-HPV (n = 43)	p*	RR; (95% CI)
Age; mean years (±SD)	36.3 (9.7)	42.1 (10.4)	0.001	0.98; (0.96–0.99)
Age ≥ 50 years, n (%)	12 (10.3)	11 (25.6)	0.014	
University education, n (%)	68 (58.1)	19 (44.1)	0.2	
Partners in the previous 12 months; median (IQR)	1 (1–5)	1 (0.75–4.25)	0.24	
Life-time partners; median (IQR)	80 (25–300)	50 (19–212.5)	0.38	
Time since commencement of sexual activity (years); median (IQR)	17 (9–24)	21 (13–27)	0.036	1.35; (1.001–1.811)
Condom use, n (%)	96 (82.1)	29 (67.4)	0.048	1.14; (0.857–1.509)
% condom use; median (IQR)	100 (70–100)	98 (0–100)	0.066	
Perianal/genital warts, n (%)	31 (26.5)	16 (37.2)	0.19	1.57; (0.466–1.716)
History of warts, n (%)	34 (29.1)	13 (30.2)	0.8	
Smoking, packets/year; median (IQR)	1.8 (0–12)	0.7 (0–16)	0.8	
	21 (17.9)	7 (16.3)	0.8	
Ex-smoker, n (%)	1 (0.85)	0 (0)	1	
Ex-IVDA, n (%)	0.14 (0–1)	0 (0–1.3)	0.66	
Duration of HIV; mean (IQR)	54 (19–118)	74 (49.2–139.7)	0.002	0.998; (0.995–1.002)
CD4 mean nadir; cells/µL (± SD)	345.9 (±217.9)	323.1 (±164.5)	0.54	
Treatment naïve	12 (10.3)	2 (4.6)	0.35	1.33; (0,99–1.77)
VL of HIV, log10 (±SD)	3.8 (±4.5)	3.4 (±4.2)	0.8	
VL <50 copies/mL, n (%)	90 (76.9)	39 (90.7)	0.051	
VL ≥200 copies/mL, n (%)	21 (17.9)	1 (2.3)	0.01	1.42; (1.172–1.732)
CD4 mean; cells/µL, (±SD)	683.7 (±263.6)	717.3 (±228.3)	0.46	
CD8 mean; cells/µL (±SD)	984.1 (±407.2)	996.9 (±402.7)	0.86	
Prior AIDS diagnosis; n (%)	33 (28.2)	11 (25.6)	0.74	
Median duration of ART; months (IQR)	28 (10–95)	60.5 (31.5–124.5)	0.08	1.002; (0.998–1.005)
	1 (1–2)	1 (1–2)	0.59	
Syphilis, n (%)	28 (23.9)	7 (16.2)	0.29	
Others STD, n (%)	22 (18.8)	7 (16.3)	0.8	
HCV, n (%)	4 (3.4)	1 (2.3)	1	
HBV, n (%)	1 (0.85)	2 (4.6)	0.49	

* p < 0.05

#### Multivariable analysis

In the multivariate analyses were applied using Poisson regression analysis., we only observed older age to be a protective factor against infection by oncogenic virus (RR: 0.97; 95% CI 0.96–0.99) and, as a risk factors the early commencement of sexual activity (RR: 1.35; 95% CI 1.001–1.811) and viral load >200 copies/mL (RR: 1.42; 95% CI 1.172–1.732) (Table 6).

## Discussion

In this clinical trial conducted in Spanish HIV-positive MSM population, no patients had grade 3–4 adverse events (AE) related to the vaccine administration. The commonest AE was local injection-site pain which was more frequent in the placebo group. In a contrasting result [22] a different (vaccine adjuvant) placebo was used, but this result is negligible. There were no changes in the HIV viral load or levels of CD4 with any of the doses used, consistent with another study [25]. Finally, in another randomized, double-blind clinical trial that compared the bivalent vs. quadrivalent vaccine (Cervarix© vs. Gardasil©, is the one we employed) in HIV-infected adults; mild injection site reactions were more common in the Cervarix© group than in the Gardasil© group (91.1% vs. 69.6%; p = 0.02) and no serious EA occurred [32], despite the fact that both had a similar adjuvant.

With respect to immunogenicity, in our study 76% of patients receiving the vaccine had measurable antibody levels at the 1th month following administration of the 3rd dose of the vaccine. Although this was significantly higher in the vaccine arm, the lack of baseline antibody levels precludes a definite conclusion that the vaccine is immunogenic. The prevalence of patients with detectable antibody against HPV is lower than in a previous clinical trial carried-out in HIV MSM patients in whom 98% of the patients developed antibodies against the four HPV genotypes in the quadrivalent vaccine [25]. These differences could result from different assay sensitivities, and there is a lack of a standardized diagnostic test to measure Ab of HPV in blood; the different study design in that trial limits direct comparison with this study; the clinical significance of antibodies following qHPV vaccination is not known. On the other hand, there is no established relationship between antibody titres and vaccine efficacy in EGL, anal intra-epithelial neoplasia, and cervix, vulva or vaginal cancer [25, 26]. The response rates in this study were lower than previously reported [33], though they were obtained in an older population and using a different assay technique. Possible causes for a lower antibody titre include prior infection not detectable by anal HPV DNA testing or serology [26], and being MSM [34].These patients may have a worse immunogenic response to the vaccine compared to heterosexuals as implied in a previous clinical trial with the nine-valent HPV vaccine [34] in which, for all HPV genotypes, the geometric mean titers at month 7 were numerically lower in MSM than in heterosexual men [34].

The prevalence of HR-HPV in the anal mucosa in our group of HIV-positive MSM patients was 73.7%. Similar percentages have been communicated in previous research in seropositive MSM [31]. However, in a population of HIV-negative MSM patients, the prevalence of HPV in anal mucosa was much lower, around 40% [35]. Our patients, similar to others who are immunocompromised, present a higher number of viruses, possibly due to the lower capacity of clearance of the virus from the anal mucosa [36].

With respect to the genotypes against which the qHPV generates immunity, in our cohort we found, in the pre-vaccination analyses, that the most frequent of all was genotype 16, which was present in 1 of every 4 patients, and only 4.5% of our patients had a simultaneous infection by two oncogenic genotypes (16 and 18). On analysing the combinations of viruses pertaining to the species 16 and 18, we observed that up to 50% of the study participants were infected by the viral species HPV16 and 37% by the species HPV18. HPV16 is the genotype that has been shown most frequently, in the majority of published studies, to be associated with ano-genital pathology in both genders [37, 38]. HIV infection is among the associated risk factors in MSM patients [39]. This low level of simultaneous infection by HPV16 and 18 of only 4.5%, and for both viruses separately >30%, implies that an important proportion of MSM seropositive patients of this cohort could benefit from the HPV vaccine. This hypothesis will be tested at the conclusion of the current, ongoing, trial. However, we must take into account that the ACTG A5298 study carried out in both seropositive men and women did not support routine vaccination of older HIV-positive adults for prevention of anal HPV infection or improving anal HSIL [26]. The main disadvantage of this clinical trial was that data concerning effectiveness of the vaccine in both women and men were analyzed together. Considering some clinical trials detected differences in the formation of antibodies between genders, being lower in older males, this could translate into lower global efficacy rates [27]. Patients in this study also had a higher median age compared to previous studies [26].

We observed that only 14.2% had HSIL lesions at V1; results similar to those communicated by other authors [31, 40], and much lower than the earlier findings of other studies in which the level was 54% [41]. These differences are possibly due to the historical period in which the researches were conducted. Not only our study but also those cited above [30, 37] were conducted in the period of late ART (after 2005) and another in the era of early ART (before 2005) [42].

In relation to the risk-factors associated with the infection of HR-HPV in the anal mucosa, multivariate analyses identified viral load ≥200 copies/mL as risk predictor for HR-HPV infection, whilst older age was protective. A study, that analysed the incidence and clearance of anal high-risk human papillomavirus in HIV-positive MSM, found that those with low HIV-RNA-viral load had the highest clearance [43]. Classically, youth has been associated with the infection of HPV in anal mucosa [8]. Data have been presented showing that, in patients >50 years of age, the virus prevalence is only 5.9% [44]. In our study patients over 50 years had a prevalence of HR-HPV infection of only 10%, being statistically significant (p = 0.014). In other hand, there are evidences that naturally acquired antibodies to HPV-16, and to a lesser extent HPV-18, are associated with some reduced risk of subsequent infection with the same HPV type [45, 46].

The principal limitations of our study that need to be highlighted derive from the exclusion criteria which do not permit generalisation of data; 18.7% of patients who underwent screening were excluded because of HSIL (14.2%) or because of simultaneous infection by genotypes 16 and 18 (4.5%). Baseline Ab were also not determined and this event is a potential weakness. The results of our study suggest immunogenicity, but without baseline differences to compare we can’t be certain.

Consequently, a further study would be necessary to demonstrate the usefulness of the vaccine in these subsets of patients who had been excluded.

## Conclusions

This trial of qHPV vaccine conducted in Spanish HIV-positive MSM patients showed significantly higher anti-HR-HPV antibody titres in vaccinated individuals than in unvaccinated controls. There were no serious adverse events attributable to the vaccine. Although current prevalence of HPV 16/18 is low, a large proportion of men likely have had incident HPV 16/18 infections and cleared them; therefore, they may have natural immunity that protect against subsequent infection. Older age was the protective factor against HR-HPV infection and HIV no suppressed was the risk factor.

## Abbreviations

qHPV vaccine: quadrivalent human papillomavirus vaccine
MSM: men having sex with men
HR-HPV: high-risk human papillomavirus
HSIL: High Squamous Intraepithelial Lesions
HIV: human immunodeficiency virus
LSIL: low squamous intra-epithelial lesion
ASCC: anal squamous cell carcinoma
HRA: high-resolution anoscopy
HM: heterosexual males
STD: Sexually Transmitted Diseases
AE: adverse events
VL: viral load
PCR: polymerase chain reaction
ART: anti-retroviral therapy
HBV: hepatitis B viral
HCV: hepatitis C viral
